# The sequence and de novo assembly of the genome of the Indian oil sardine, *Sardinella longiceps*

**DOI:** 10.1038/s41597-023-02481-9

**Published:** 2023-08-25

**Authors:** Sandhya Sukumaran, Wilson Sebastian, A. Gopalakrishnan, Oommen K. Mathew, V. G. Vysakh, Prathibha Rohit, J. K. Jena

**Affiliations:** 1 ICAR-Central Marine Fisheries Research Institute, Ernakulam North P.O., Kochi, Kerala 682018 India; 2 Agrigenome Labs Pvt. Ltd., Kakkanad, Kochi, Kerala 682042 India

**Keywords:** Genome, Genomics

## Abstract

The Indian oil sardine, *Sardinella longiceps*, is a widely distributed and commercially important small pelagic fish of the Northern Indian Ocean. The genome of the Indian oil sardine has been characterized using Illumina and Nanopore platforms. The assembly is 1.077 Gb (31.86 Mb Scaffold N50) in size with a repeat content of 23.24%. The BUSCO (Benchmarking Universal Single Copy Orthologues) completeness of the assembly is 93.5% when compared with Actinopterygii (ray finned fishes) data set. A total of 46316 protein coding genes were predicted. *Sardinella longiceps* is nutritionally rich with high levels of omega-3 polyunsaturated fatty acids (PUFA). The core genes for omega-3 PUFA biosynthesis, such as Elovl 1a and 1b,Elovl 2, Elovl 4a and 4b,Elovl 8a and 8b,and Fads 2, were observed in *Sardinella longiceps*. The presence of these genes may indicate the PUFA biosynthetic capability of Indian oil sardine, which needs to be confirmed functionally.

## Background & Summary

The Indian oil sardine, *Sardinella longiceps* is a small pelagic fish occurring along coastal shelf waters at depths of 20–200 m. It is distributed mainly along the north-east, south-east, south-west and north-west Indian coasts, the Gulf of Oman and the Gulf of Aden^1^. *Sardinella longiceps* is one of the most important fisheries resources of the Indian subcontinent and makes the largest economic contribution (about 10%) to the total marine fisheries of India^2^. Sardines are also utilized as a raw material for manufacture of fish meal^3^. They are ecologically important as they form an intermediate link in the trophic network as a planktivore which is preyed upon by larger predators^4^. Small pelagic fishes like the Indian oil sardines can be considered as model organisms to study the climatic and fishing impacts on the Indian Ocean resources, as they respond to alterations in environmental and oceanographic parameters with localized extinction and recolonization and possible cascading effects at trophic levels^5^. The fishery of this species peaks around the Malabar upwelling zone of the western Indian Ocean upwelling system^6,7^ and the fishery exhibited high variability on a decadal scale, with periods of abundance and crashes during this century^8,9^. A comprehensive investigation of its population genetic structure, selection and adaptive variation has been carried out by the present authors^10–13^, revealing the presence of genetic structuring and local adaptation. The adaptation patterns have also been linked to the environmental and oceanographic characteristics of the Indian Ocean^13^. Genetic and genomic investigations in Indian oil sardine^10–13^ revealed the presence of two highly differentiated stocks *viz*., Indian and Gulf of Oman stocks. The whole genome data will be valuable to understand the genomic rearrangements and polymorphisms specific to Indian oil sardine populations which could further be linked to the environmental and oceanographic conditions of the Northern Indian Ocean. The whole genome data forms a great resource for formulating management measures for the conservation and sustainable utilization of the Indian oil sardine. The Indian oil sardine constitutes a trans-boundary resource and the whole genome information can also be utilized for certification of the fishery and identification of the origin of catch for monitoring clandestine trade mainly in the fishmeal industry.

Rich in polyunsaturated fatty acids (PUFA), protein and essential vitamins, *S. longiceps* provides a cost-effective source of high-quality protein and essential fatty acids for millions of people, particularly in developing countries like India^14,15^. Long-chain polyunsaturated fatty acids (LC-PUFA) such as eicosapentaenoic acid (EPA; 20:5n-3) and docosahexaenoic acid (DHA; 22:6n-3) play important roles in several physiological functions like nerve development, anti-inflammatory effects and cardiovascular health^16^. They also play an important role in gene regulation as ligands of transcription factors, and are important for cell membrane structure and lipid signaling^17,18^.

*Sardinella longiceps* contains more n-3 PUFAs than n-6 PUFAs^14^ and DHA and EPA contribute to the n-3 PUFA composition along with low levels of linolenic acid (<2%). *Sardinella longiceps* is also considered as a high-fat fish with muscle lipid content greater than 8%. The lipid storage sites in fishes are located in the subcutaneous tissues, muscle tissue, belly flab, liver, mesenteric tissue and the head^14^. Lipids and their constituent fatty acids, together with proteins, are the main organic components of fish and constitute the main sources of metabolic energy for growth, reproduction, movement and migratory activities^19^.

Vertebrates acquire LC-PUFAs mainly through their diets. LC-PUFAs can also be biosynthesized endogenously from shorter PUFAs mainly linoleic acid (LA;18:2n-6) and α-linolenic acid (ALA;18:3n-3) through a series of elongation and desaturation reactions^20,21^. However, the ability to biosynthesize PUFAs from LA and ALA endogenously varies among species and this ability is more pronounced in freshwater fish than in marine fish^19^. The differential ability to biosynthesize PUFAs is mainly attributed to the fatty acid rich diet of marine species, causing repression of endogenous de novo biosynthesis of fatty acids and chain elongations^19^.

The two important enzymes involved in the biosynthesis of long-chain polyunsaturated fatty acids (LC-PUFAs) are elongases (Elovls) and fatty acid desaturases (Fads)^22^. Elovls are considered as the initial and rate-limiting enzymes that participate in the elongation reaction required for the de novo biosynthesis of LC-PUFA. The Elovls family has Elovl 1–8 of which Elvol2, Elovl4, Elovl5 and Elovl8 are involved in the elongation of LC-PUFA^23–25^. Elovl2 is presumed to be preferentially involved in the elongation step from C22 to C24 LC-PUFA^26^. Recent investigations indicated the successful characterization of the Elovl genes in teleosts^22^. Elovl2, Elovl4 (with paralogues Elovl 4a and Elovl 4b), Elovl5 and Elovl8 (with paralogues elovl8a and elovl8b) have been characterized from teleosts, contradicting previous reports of the lack of PUFA biosynthetic capability in marine fish^22^. Fatty acid desaturases enzymes catalyze the insertion of new double bonds (unsaturations) into Mono Unsaturated Fatty Acids (MUFAs)^27^. Genes encoding desaturase enzymes in vertebrates include Fads1 and Fads 2, which encode Δ5 and Δ6 desaturases respectively^22^.

*Sardinella longiceps* is a species with high omega-3 PUFA content and hence we investigated the type of Elovls and Fads genes in *Sardinella longiceps*. We also made a comparative analysis with the closely related andaromous Hilsa shad, *Tenualosa ilisha*.

The diploid chromosome number of *Sardinella longiceps* is 48 (2n) and the chromosomes are acrocentric in shape^28^. We estimated the genome size of *S. longiceps* as 1.25 Gb based on flow cytometry analysis. The whole genome of the Indian oil sardine, *S. longiceps*, was characterized by adopting an integrated approach using Illumina and Nanopore technologies. High quality data were generated for assembly and annotation. Further, we also identified the genes involved in PUFA biosynthesis in the Indian oil sardine, *S. longiceps* and the closely related anadromous shad, *Tenualosa ilisha*. We performed a phylogenetic analysis based on single copy genes of *S. longiceps* and 13 other species belonging to the ray-finned fish (Actinopterygii) taxa. The genome assembly of Indian oil sardine forms an important genomic resource for further studies on adaptive variation and selection at the genome level in the face of climate change in pelagic fishes distributed across wide environmental clines. In addition, the genomic machinery that contributes to high nutritional quality could also be studied.

## Methods

### Sample collection

**Fig. 1:**
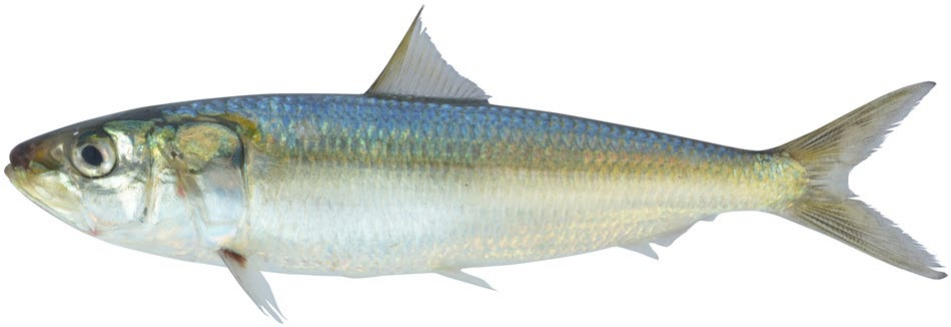
A photograph of the Indian oil sardine, *Sardinella longiceps* used for whole genome sequencing.

An adult male specimen of *S. longiceps* was collected live from the local fishery off Kochi, Kerala, India (Fig. 1). The fish was anesthetized using 2-phenoxy ethanol (1:250 v/v), and killed by cervical section. The muscle tissues were flash frozen in liquid nitrogen and stored at −80 °C until DNA extraction. Additionally, the heart, gonad, and liver of the same individual were dissected out into RNA later for transcriptome sequencing and stored at −80 °C until RNA extraction. Fish collected for this purpose was handled in accordance with the guidelines for the care and use of fish in research by De Tolla *et al*.^29^. Further, these protocols were approved by the Ethics Committee of ICAR-Central Marine Fisheries Research Institute, Kochi (Approval No: MBT/GEN/25-01).

### DNA extraction and genome sequencing

Extraction of genomic DNA was carried out from muscle tissue using a genomic DNA isolation kit (PureLink Genomic DNA Mini Kit, Invitrogen) according to the manufacturer’s protocol. Libraries were constructed for subsequent sequencing on Illumina Hiseq 2500 (Illumina Inc., San Diego, CA, USA) and PromethION (Oxford Nanopore Technologies, Oxoford, UK) systems using the isolated DNA. Paired-end libraries with an insert size of 500 bp were prepared using the NEBNext Ultra DNA Library Prep Kit (NEB) and mate pair libraries with insert sizes of 270 bp, 500 bp and 700 bp were prepared using the Nextera Mate Pair Library Prep Kit (NEB) following Illumina standard procedure. The paired-end (PE) and mate-pair (MP) libraries were then sequenced (100X coverage for PE and 60X for MP) on the HiSeq 2500 System in 150 bp PE mode and 250 bp PE mode, respectively. For Nanopore libraries (35X coverage), high molecular weight gDNA was size-selected (1040 kb) with the Blue Pippin system (Sage Science, Beverly, USA) and was processed using ligation sequencing gDNA kit (Oxford Nanopore Technologies, Oxford, UK) following manufacturer’s instructions, and sequenced on PromethION system.

We generated 113.22 Gb of raw reads using paired-end sequencing with a read length of 150 bp and also approximately 13.35 Gb of raw reads from mate-pair libraries with a read length of 250 bp. The fastq files were pre-processed by adapter removal and filtering out the reads with an average quality score of less than 30 in any of the paired end reads using Trimmomatic v0.39^30^. Approximately 100 Gb of clean paired end reads and 10 Gb of mate pair reads were retained for further assembly. Of the generated 36.14 Gb raw nanopore reads, 30 Gb reads with a mean length of 20 kb passed quality control, after removing low-quality reads with a mean_q score of <7. Nanopore reads were subsequently corrected by mapping the clean Illumina reads to the Nanopore sequence data using the LoRDEC32 program with default parameters^31^.

### RNA extraction and transcriptome sequencing

Muscle, heart, gonad and liver tissues of *S. longiceps* were dissected out and total RNA was extracted from each tissue using Trizol reagent (Invitrogen) and treated with DNase I to remove genomic DNA. The integrity of the sample was confirmed using a Bioanalyzer (Agilent 2100) and RNA extracted from all tissues was pooled at equimolar concentration. RNA library preparation was performed with NEBNext Poly(A) mRNA magnetic isolation module kit (NEB) and NEBNext Ultra RNA library preparation kit (NEB) following manufacturer’s protocol and sequenced using Illumina HiSeq 2500 paired-end 150 base pair cycle. A total of 21 Gb of data was generated, which was then used for transcriptome identification and genome annotation.

### Estimation of the genome size

The genome size of Indian oil sardine, *Sardinella longiceps* was estimated using flow cytometry. Flow cytometry analysis of genome size involves staining the DNA of individual cells using propidium iodide^32^ or DAPI^33^ and analysis of fluorescence. Flow cytometry is considered to be more accurate than other methodologies^34^. Blood samples were collected from 5 individuals of Indian oil sardine after anesthetizing the fishes with 2-phenoxyethanol (Sigma-Aldrich, USA). The blood was collected from the caudal vein using 5 ml syringe containing 0.01 M phosphate buffered saline (PBS). The blood cells were centrifuged at 5000 rpm for 8 min to precipitate the blood cells. The blood cells (precipitate) were washed with 0.01 M PBS and fixed in ice cold 70% ethanol at 4 °C. Propidium iodide (PI) staining was carried out after washing the cells twice with 0.01 M PBS and removing RNA by adding DNase free RNase A (Qiagen, Germany)^33^. The samples were then filtered through sterile cell strainer of 40 µm (Corning, Sigma-Aldrich, Co., St. Louis, Mo, USA) and analysed using flowcytometer. Chicken red blood cells (RBCs) were used as standard and processed similarly. The genome size of the Indian oil sardine, *Sardinella longiceps* was estimated using a Beckman Coulter Cytoflex flow cytometer with laser excitation at 488 nm and a minimum of 10,000 events (cells) per sample. The genome size was estimated at 1.25 Gb.

### ***De Novo*** genome assembly

The genome was assembled following a hybrid strategy of combining both clean Nanopore and Illumina reads using the Flye assembler 2.9.1^35^ based on the automatic minimum overlap option. The initial assembly was then polished in POLCA^36^ using Illumina reads. The polished assembly was compared to the NCBI NT database using the BLASTx program^37^ with an E-value cutoff of 10^−5^ using OmicsBox software and the contigs with the best BLASTx hit based on query coverage, identity, similarity score and description were filtered out. Contigs matching the taxonomy lineage Vertebrata were extracted, resulting in 17447 contigs. The filtered contigs were scaffolded (Reference guided scaffolding) using Ragoo^38^ using the *Clupea harengus* (https://ftp.ncbi.nlm.nih.gov/genomes/all/GCF/900/700/415/GCF_900700415.2_Ch_v2.0.2/GCF_900700415.2_Ch_v2.0.2_genomic.fna.gz) genome as reference. The final assembly resulted in a genome of 1077 Mb in size with a scaffold N50 of 31.86 Mb. Assembly statistics are given in Table 1. The completeness of the assembly was evaluated using BUSCO assessment with BUSCO v5.3.2^39^. A total of 3400 out of the 3640 (93.5%) of the Actinopterygii gene set (Actinopterygii_odb10) were fully identified in the assembled genome. The genome module benchmark values were calculated as C: 93.5%, including S: 86.5%, D: 7.0%, F: 3.0%, M: 3.5% and n = 3640 (C: complete, S: single-copy, D: duplicated, F: fragmented, M: missing and n: total BUSCO groups of Actinopterygii_odb10 data).

**Table 1:** Statistics of the assembled genome of Indian oil sardine, *Sardinella longiceps*.

Genome assembly statistics	Data	
Total length	1,077,164,011	
Number of scaffolds	10,318	
Longest scaffold	43,849,268	
N50 scaffold length	31,865,965	
GC rate (% of genome)	43%	
Repeat elements (% of genome)	23.24%	
**BUSCO genome completeness score**	**Data**	**Ratio**
Complete BUSCOs	3400	93.5%
Complete and single copy BUSCOs (C)	3147	86.5%
Complete and duplicated BUSCOs (D)	253	7.0%
Fragmented BUSCOs (F)	108	3.0%
Missing BUSCOs	132	3.5%
Total number of Actinopterygii orthologs	3640	

### ***De Novo*** transcriptome assembly

The fastq files were pre-processed before performing the assembly. Adapter removal and quality trimming was carried out using Trimmomatic v0.39^30^ with quality cut off Q30. Further, the rRNAs were removed by aligning with the SILVA database^40^. The cleaned reads were assembled using Trinity v2.14.0^41^ with default settings and generated 95,426 transcripts. Similar sequences were clustered using CD-HIT-EST^42^ to remove redundant sequences. We found alignment coverage (alignment length to transcript length) of 72% for expressed genes in the genome assembly.

### Repeat annotation

Repetitive elements were detected in the genome of Indian oil sardine using *ab initio* prediction and homology annotation. LTR FINDER^43^, RepeatModeler (http:// https://www.repeatmasker.org/RepeatModeler)^44^ and RepeatScout^45^ were used with default parameters to detect various types of repeat elements. Further, RepeatMasker (https://www.repeatmasker.org/)^46^ was used to construct a new repeat elements library based on the Repbase TE v21.01. Tandem elements were identified using the Tandem Repeats Finder. Repeat Masker and Repeat ProteinMask were used with default parameters to identify known repeat element types against the Repbase database. A total of 250.29 Mb of repetitive elements were identified in the genome of the Indian oil sardine, accounting for 23.24% of the assembled *S. longiceps* genome (Table 2). The repeat content is nearer to that of the European sardine, *Sardina pilchardus* (23.33%)^47^ and lower than that of the Atlantic herring, *Clupea harengus* (30.9%)^48^.

**Table 2:** Statistics of repeat elements in the genome of *Sardinella longiceps*.

Repeat Classes	Number of Elements	Length	Percentage of genome
**Retroelements**	**632,667**	**65,430,438 bp**	**6.07**
1. SINEs	18,359	2,053,583 bp	0.19
2. LINEs	178,218	25,491,898 bp	2.37
3. LTR elements	436,090	37,884,957 bp	3.52
**DNA transposons**	**2,386,246**	**172,941,995 bp**	**16.06**
**Unclassified**	**162,650**	**11,927,206 bp**	**1.11**
**Total interspersed repeats:**		**250,299,639 bp**	**23.24**

### Protein coding gene prediction and functional annotation

Gene predictions were performed using *ab initio*, homology-based and transcriptome based prediction strategies. All of these predictions were made using the AUGUSTUS gene prediction server (https://bioinf.uni-greifswald.de/augustus/) through the OmicsBox Version 2.2 platform (https://www.biobam.com/omicsbox/) using *ab initio* and extrinsic evidence options. The repeat -masked sequences were used as input for *ab initio*, homology and transcriptome based predictions. Homology based predictions were made using the proteome data of *Clupea harengus, Sardina pilchardus, Danio rerio, Takufugu rubripes, Oryzias latipes* and *Salmo salar*. A final non-redundant gene set was generated by merging all the gene sets from these three approaches using MAKER^49^. The homology search was performed using the BLASTx utility^50^ with an E-value threshold of 1E-5. Functional annotations were performed for the combined gene set generated through all the prediction strategies via OmicsBox using biological databases; Uniprot (https://www.uniprot.org/), KEGG pathways and EggNOG databases^51^. Gene ontology annotations were performed by the InterProScan program^52^. A total of 46316 protein-coding genes were predicted with a mean length of 1851 bp. About 44279 (95.6%) of the total predicted genes were assigned with function annotation. The BUSCO completeness of the annotation was 86.65%, S: 78.65%, D: 8%, F: 4.62%, M: 8.74% and n = 3640 (C: complete, S: single-copy, D: duplicated, F: fragmented, M: missing and n: total BUSCO groups of Actinopterygii_odb10 data).

### Ortholog and phylogenetic analyses

**Fig. 2:**
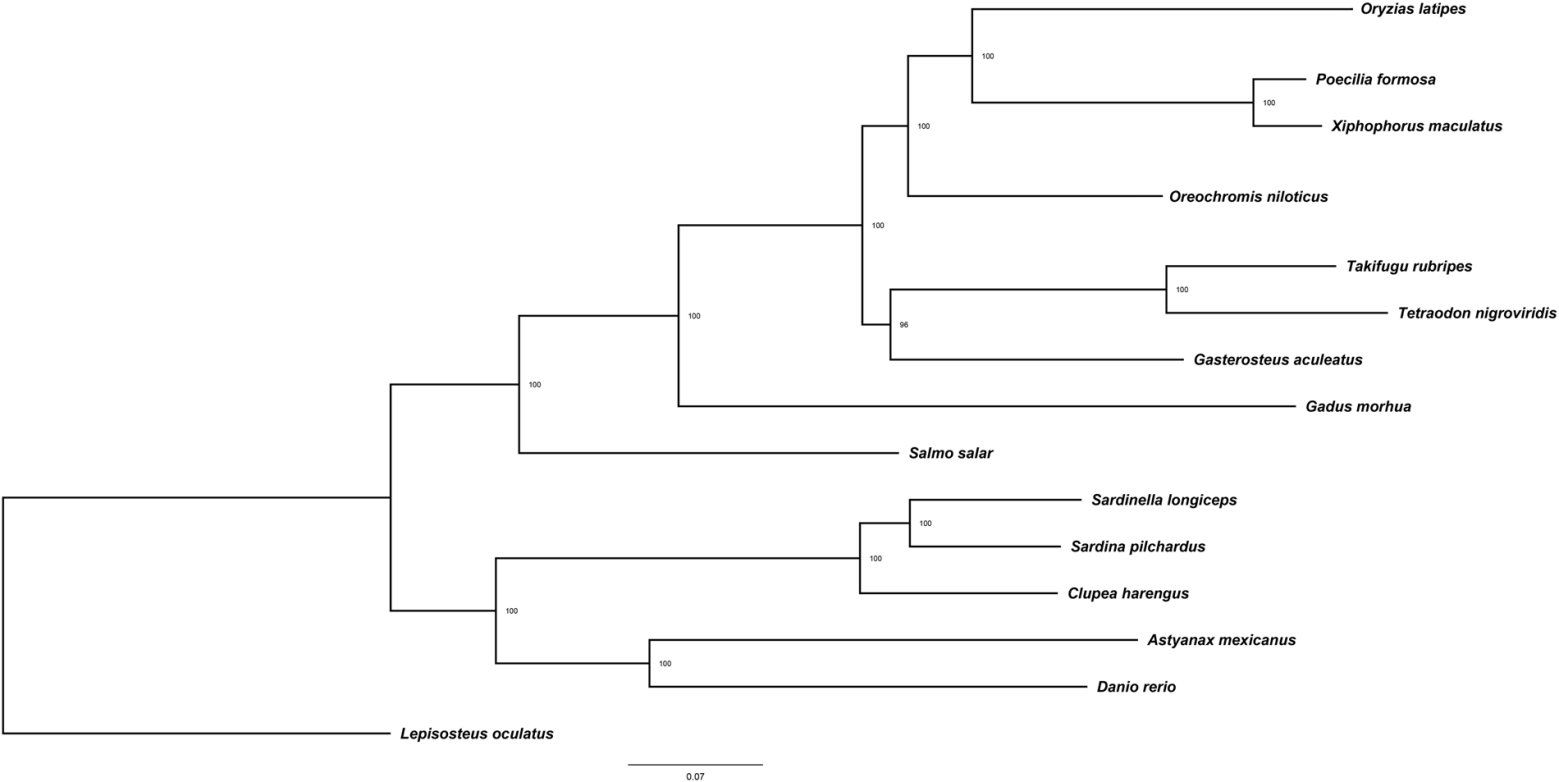
Maximum likelihood phylogenetic tree generated using single copy orthologous genes from 13 representative teleosts and *Sardinella longiceps*. *Lepistosteus aculeatus* was used as an outgroup. The tree was generated using IQ-TREE v 2.1.4.

Reference protein sequences of 14 representative species including Atlantic herring (*Clupea harengus*), European pilchard (*Sardina pilchardus*), Japanese rice fish (*Oryzias latipes*), Amazon molly (*Poecilia formosa*), Southern platy fish (*Xiphophorus maculatus*), Nile Tilapia (*Oreochromis niloticus*), Japanese puffer (*Takifugu rubripes*), Green spotted puffer (*Tetraodon nigrovidis*), Three spined stickle back (*Gastrosteus aculeatus*), Atlantic cod (*Gadus morhua*), Atlantic salmon (*Salmo salar*), Mexican tetra (*Astyanax mexicanus*) and Zebra fish (*Danio rerio*) were downloaded from Ensembl (https://www.ensembl.org) and NCBI (https://www.ncbi.nlm.nih.gov/) databases. The protein sets were filtered by removing protein sequences with less than 50 amino acids. These sequences, along with the *S. longiceps* protein set, were used to identify orthologous genes with OrthoFinder v 2.5.4 (-S diamond -I 1.5 -M msa -A mafft -T fasttree -oa)^53^. Phylogenetic analyzes were performed by aligning the single-copy orthologous genes from all species and concatenating the alignments species-wise. A Maximum Likelihood (ML) tree was constructed based on these alignments using IQ-TREE v 2.1.4 (--seqtype AA -m JTT + F + I + G4 -bb 10000 -alrt 10000)^54^ (Fig. 2). Species belonging to the family Clupeidae, the Indian oil sardine, *Sardinella longiceps*, and European pilchard, *Sardina pilchardus* clustered in the same clade, while the Atlantic herring, *Cluepa harengus* diverged into a separate but closely related clade. The phylogenetic tree corroborated the findings from traditional taxonomy.

### Identification of omega-3 PUFA biosynthesis related genes

**Fig. 3:**
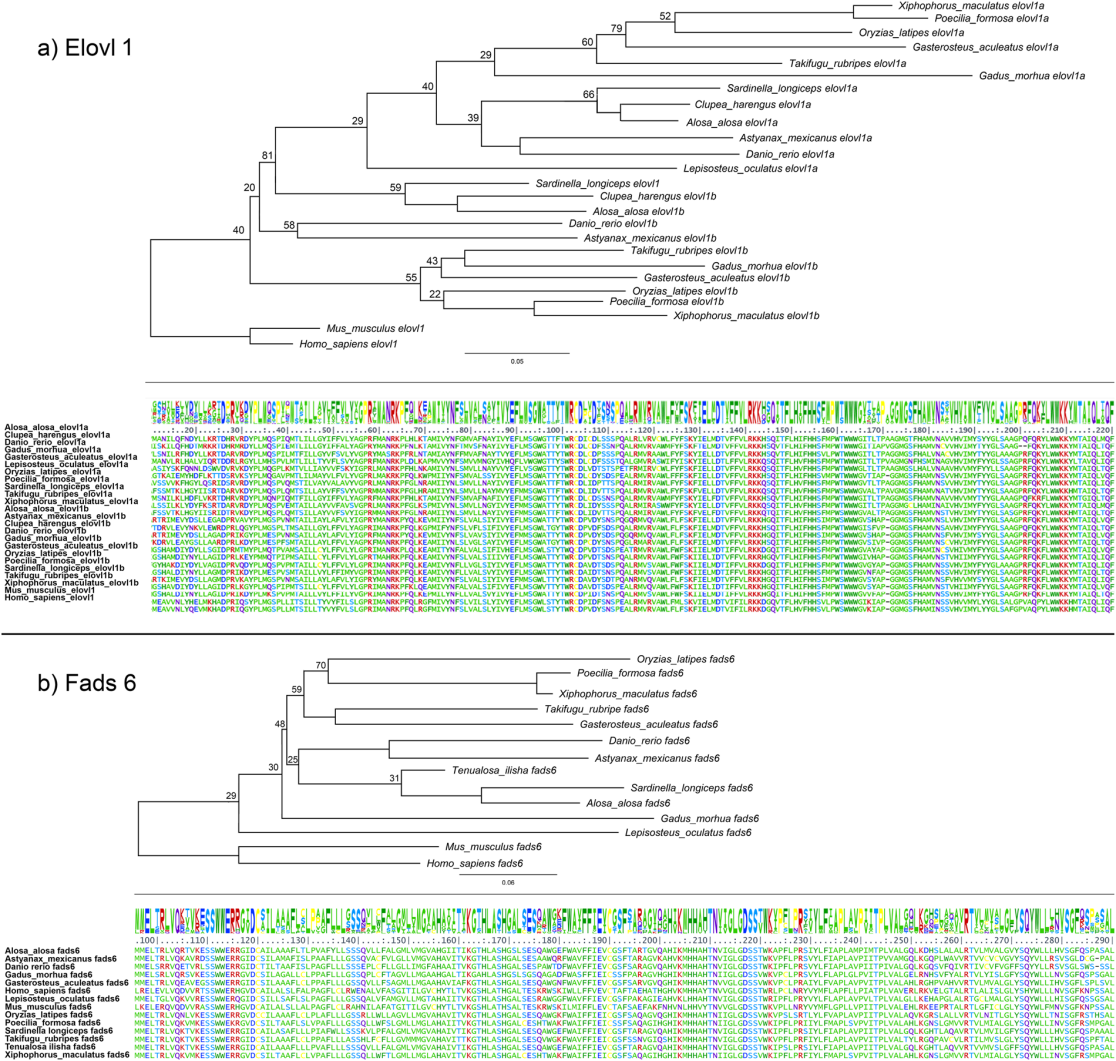
(**a**) Comparison of the Elovl 1 protein of *Sardinella longiceps* with selected species and maximum likelihood phylogenetic tree constructed using these sequences (**b**) Comparison of Fads 6 protein of *Sardinella longiceps* with selected species and maximum likelihood phylogenetic tree constructed using these sequences. The tree was generated using IQ-TREE v 2.1.4.

The key gene families involved in omega-3 PUFA biosynthesis viz., elongases (*Elovl*) and desaturases (Fads) reported from fishes were identified using OrthoFinder v 2.5.4 (-S diamond -I 1.5 -M msa -A mafft -T fasttree -oa)^53^ and were used as the queries to align against *S. longiceps* genome using TBLASTn^55^. GeneWise^56^ was then used to predict gene structures based on these alignment. We also predicted the omega-3 PUFA biosynthesis genes from the genome of *Tenualosa ilisha*, a closely related anadromous shad^57^. The core genes for omega-3 PUFA biosynthesis in the *S. longiceps* were, Elovl 1a and 1b,Elovl 2, Elovl 4a and 4b and Elovl 8a and 8b. In contrast, all Elovl genes (Elovl1a and 1b,Elovl2, Elovl3, Elovl4a, Elovl5, Elovl6, Elovl7a, Elovl8a and 8b) were found in the genome of the closely related anadromous clupeid, *Tenualosa ilisha*. Elovl 1, 3, 6 and 7 are presumed to be involved in SFA (Saturated Fatty Acids) and MUFA (Mono-unsaturated Fatty Acids) formation whereas Elovl2, Elovl4, Elovl5 and Elovl8 are important for PUFA biosynthesis^22^. Among the desaturases, only Fads2 (∆6 desaturase) was present in both *S. longiceps* and *T. ilisha*. The presence of Elovl2, Elovl4, Elovl8 and Fads 2 in *S. logiceps* may be an indication of the PUFA biosynthetic capability which needs to be confirmed by functional characterization. A comparison of the Elovl 1 and Fads 6 proteins of *Sardinella longiceps* with selected species is given in Fig. 3. Phylogenetic analyzes were performed by aligning the omega-3 PUFA biosynthesis genes from selected species. A Maximum Likelihood (ML) tree was constructed based on these alignments using IQ-TREE v 2.1.4^54^ (Fig. 3; tree corresponding to Elovl 1 and Fads 6 shown).

## Data Records

The genome assembly of *S. longiceps* has been deposited with NCBI, GenBank, under accession number JAODXP000000000.1^58^ (contigs; JAODXP010000001-JAODXP010010325), BioProject ID: PRJNA873888 and BioSample ID: SAMN30503998. The transcriptome sequence dataset has been deposited in the Sequence Read Archive (SRA) under project number SRR21289080^59^. The DNA sequence dataset generated from ONT PromethION sequencing were deposited under project number SRR21289081^60^. The DNA sequence dataset generated from Illumina HiSeq 2500 (mate pair library) was deposited under project number SRR21289082^61^. The DNA sequence dataset generated from Illumina HiSeq 2500 (paired end library) was deposited under project number SRR21289083^62^. The files of the assembled genome and annotation of *S. longiceps* were deposited in Figshare database under DOI code^63^.

## Technical Validation

The completeness of the *S. longiceps* genome assembly was assessed using BUSCO v5.2.2. and 93.5% of the BUSCO genes were complete.

## Data Availability

The genome and transcriptome analyses were performed following the manuals and protocols of the cited bioinformatic software. No new codes were written for this study.
